# Improving Care for Veterans Through Health Equity Research

**DOI:** 10.1089/heq.2023.0017

**Published:** 2023-05-26

**Authors:** Rachel Ramoni, Carolyn Clancy

**Affiliations:** ^1^ Office of Research and Development, Department of Veterans Affairs, Washington, District of Columbia, USA.; ^2^ Discovery, Education and Affiliate Networks, Department of Veterans Affairs, Washington, District of Columbia, USA.

Veterans are a diverse population of individuals united by their service to our country. In addition to providing health care to Veterans, Veterans Affairs (VA) also has a mission to conduct research to improve Veterans' and indeed our nation's well-being. To succeed in that mission, we recognize that we must actively promote diversity, equity, and inclusion (DEI) across the full range of domains, including the research workforce, representation in research, and funding DEI research.

VA is committed to serving all Veterans, and we are honored to write the introductory column for this special edition of *VA Health Equity Research*. It represents the efforts of a team of people who are passionate about improving Veterans' well-being through actions to enhance DEI. We lost one of those team members, the late Mitchell Mirkin, far too soon. Mitch was the acting director of VA Research Communications, and he was instrumental to the conception of this special collection. This issue is dedicated to his memory.

Research across the translational continuum requires diversity in skill sets, experiences, and perspectives. There are many benefits that flow from a diverse health-equity-focused VA research enterprise: fostering scientific innovation, contributing to VA's robust learning environments, improving the equity and quality of research, and increasing the likelihood that underrepresented Veteran populations participate in and benefit from health research.

For these reasons, and many more, we are excited for the opportunity to highlight examples of health equity research conducted by VA investigators, as well as DEI efforts by the VA community.

In this issue, Dr. Suma Muralidhar, director of the VA Million Veteran Program (MVP), and colleagues describe efforts to support diversity and representation in VA research. With >912,000 enrollees, MVP is committed to expanding the participation of women Veterans. In 2022, MVP's first digital women's campaign resulted in a 54% increase in the number of women Veterans enrolled in the program. MVP resources have sparked many significant research projects. For example, Dr. Shiuh-Wen Luoh and colleagues from the VA Portland Health Care System describe their use of MVP data to develop breast cancer risk scores for women Veterans of African American ancestry.

We also highlight key DEI initiatives carried out by VA research. Our colleagues in the Veterans Health Administration Office of Health Equity, led by Dr. Ernest Moy, illustrate VA's efforts to combat the COVID-19 pandemic by addressing structural disparities. Dr. Charles Michael Hart describes the Atlanta VA Health Care System–Morehouse School of Medicine Partnered Core Recruiting Site. And two articles highlight the VA Office of Research and Development's (ORD) efforts to diversify the VA Research workforce through mentored supplement awards: a first-person perspective of ORD's Research Supplements to Promote Diversity program, and a discussion on racial differences in vascular assessments by DEI research supplement awardee Dr. Olamide Alabi and her mentors. These programs are vital to attracting the best and brightest early-career researchers to the VA research enterprise.

Finally, we would like to introduce a series of articles that cover the wide-ranging effects of health care disparities. An article written by Dr. Bharati Prasad, and colleagues, presents clinical research on racial and socioeconomic drivers of disparities in sleep health. Dr. John Blosnich explores important issues surrounding disclosure of health data in research for sexual- and gender-minority Veterans. And Dr. Leslie Hausmann and coauthors highlight just how important it is to include equity into quality improvement efforts in an integrated health care system.

We hope that you enjoy exploring this special issue as much as we did.

## Abbreviations Used

DEI: diversity, equity, and inclusion
MVP: Million Veteran Program
ORD: Office of Research and Development
VHA: Veterans Health Administration

